# Orofacial clefts in newborns in Brazil: a time series study, 2010-2021

**DOI:** 10.1590/S2237-96222025v34e20240027.en

**Published:** 2025-05-12

**Authors:** Karoline Machado Vieira, Verônica Canarim Menezes, Augusto César Cardoso-dos-Santos, Ana Luiza Meneguci Moreira Franco, Flavia Martinez de Carvalho, Lavinia Schuler Faccini, Betine Pinto Moehlecke Iser

**Affiliations:** 1 Universidade do Sul de Santa Catarina, Curso de Medicina, Tubarão, SC, Brazil; 2 Ministério da Saúde, Departamento de Ciência e Tecnologia, DF, Brazil; 3 Universidade Federal do Rio de Janeiro, Programa de Pós-Graduação em Genética, Rio de Janeiro, RJ, Brazil; 4 Instituto Oswaldo Cruz, Laboratório de Epidemiologia de Malformações Congênitas, Rio de Janeiro, RJ, Brazil; 5 Universidade Federal do Rio Grande do Sul, Programa de Pós-Graduação em Genética e Biologia Molecular, Porto Alegre, RS, Brazil; 6 Universidade do Sul de Santa Catarina, Programa de Pós-Graduação em Ciências da Saúde, Tubarão, SC, Brazil

**Keywords:** COVID-19, Cleft Lip, Cleft Palate, Time Series Studies, Brazil, COVID-19, Labio Leporino, Fisura del Paladar, Estudios de Series Temporales, Brasil

## Abstract

**Objective:**

To analyze distribution of orofacial cleft cases (cleft palate, cleft lip and cleft palate with cleft lip) and their temporal trend in Brazil, according to the country’s regions and Federative Units from 2010 to 2021, in addition to comparing proportions during the COVID-19 pandemic, from 2020 to 2021, with the preceding time series, from 2010 to 2019.

**Methods:**

This is an epidemiological time series study, using records of all babies born with orofacial clefts held on the Live Birth Information System for the period 2010-2021. Prevalence rates were calculated according to year, regions and Federative Units. Time series analysis was performed using the Prais-Winsten generalized linear model.

**Results:**

A total of 34,564,430 live births were recorded in the period. National prevalence of orofacial clefts was 6.73/10,000 live births (95% confidence interval [95%CI 6.64; 6.81])). The Southern region had the highest rate in the period for all types of orofacial clefts. The Northeast region and the states of Alagoas and Piauí showed a rising trend in the period for the three types of orofacial clefts. Other regions showed a stationary trend or increases/decreases in just one type of cleft. Comparing the pre-pandemic period with the pandemic period, there were no significant changes in the prevalence in the Brazilian regions.

**Conclusion:**

Among the country’s regions, for all three types of clefts, the South had the highest prevalence, and the Northeast had a rising trend. Among the Federative Units, there was an increase in the three types of clefts in Alagoas and Piauí. The COVID-19 pandemic did not influence prevalence in the period analyzed.

## Resumen

**Objetivo:**

Analizar la distribución de casos y la tendencia temporal de las fisuras orofaciales (fisura del paladar, labio leporino y fisura labiopalatina) en Brasil, según regiones y unidades de la Federación, en los últimos 12 años, además de comparar proporciones durante la pandemia de COVID-19, de 2020 a 2021, con la serie histórica anterior, de 2010 a 2019.

**Métodos:**

Se trata de un estudio epidemiológico de series temporales, utilizando registros de todos los bebés nacidos con fisuras orofaciales, del Sistema de Información de Nacidos Vivos, en el período 2010-2021. Las prevalencias se calcularon según año, regiones y unidades de la Federación. El análisis de series temporales se realizó mediante el modelo lineal generalizado de Prais-Winsten.

**Resultados:**

Se registraron 34.564.430 nacidos vivos en el período. La prevalencia nacional de fisuras orofaciales fue de 6,73/10 mil nacidos vivos (intervalo de confianza del 95% 6,64; 6,81). El Sur tuvo la tasa más alta en el período para todos los tipos de fisuras orofaciales. La región Nordeste y las Unidades de la Federación de Alagoas y Piauí mostraron tendencia creciente en el período en los tres tipos de fisuras orofaciales. Otras regiones mostraron una tendencia estacionaria o aumentos/disminuciones en un solo tipo de fisura. Comparando el período prepandemia con el período pandémico, no hubo cambios significativos en la prevalencia de las regiones brasileñas.

**Conclusión:**

Entre las regiones de Brasil, en los tres tipos de fisura, el Sur presentó la mayor prevalencia y el Nordeste, una tendencia creciente. Entre las unidades de la Federación, Alagoas y Piauí presentaron aumento en los tres tipos de fisuras. La pandemia de COVID-19 no influyó en las tasas de prevalencia en el período analizado.

## Introduction

Orofacial clefts are congenital craniofacial abnormalities that occur due to defects in the formation of the primary and secondary palates at the beginning of the embryo’s formation, affecting, to a varying extent, the lips and oral and nasal cavities (1). Considering the embryonic and pathogenic processes involved, they are divided into isolated cleft palate and cleft lip with or without cleft palate and can occur in isolation (70.0% of cases) or associated with a wide range of syndromes (30.0% of cases) (1-3). 

Global incidence of orofacial clefts is 1.50 per 1,000 live births, i.e. approximately 220,000 births per year, and varies according to geographic region, ethnic origin, socioeconomic level and the nature of the cleft itself (4-6). In Brazil, between 2010 and 2019, the prevalence rate was 6.20/10,000 live births, with differences between the country’s Federative Units and regions (2). Prevalence rates of around 4.85 and 5.16/10,000 live births have been found, but without analysis of temporal trend for each type of orofacial cleft (6-8).

Orofacial clefts can cause harm to teeth, appearance, speech, hearing, cognition and psychosocial aspects (1,2,9). These clefts can be prejudicial to the nutritional status of the newborn, as they influence their natural ability to feed and their sucking and swallowing mechanisms (2,10,11). It has been found that, in infants with orofacial clefts, the likelihood of mortality was 2.07 times greater than for those without this congenital abnormality (12). The impacts of orofacial clefts require multidisciplinary care, including clinical and surgical care, from birth to adulthood, as they can lead to lasting adverse outcomes for health and social integration (2).

The etiology of orofacial clefts is complex and multifactorial with genetic and environmental aspects involved in their formation (1,3). Consumption of alcoholic beverages, exposure to tobacco smoke (actively or passively) and exposure to phenytoin, valproic acid and thalidomide, in the first trimester of pregnancy, are the main environmental factors associated with congenital abnormality (1,2,12). Occurrence of maternal fever and infections has been associated with the development of orofacial clefts, as have stressful events during life and the periconceptional period and the risk of orofacial clefts in offspring (13,14).

It has been questioned whether stressful events and other infections, such as COVID-19, a disease caused by the SARS-CoV-2 coronavirus, could influence the occurrence of orofacial clefts, as they also generate a great psychological and stressful effect, in addition to symptoms that can resemble a common cold, with the presence of fever (15). Occurrence of orofacial clefts may be associated with maternal exposure to lifelong stress and fear of COVID-19 in particular, but without a direct effect of infection itself (16). The importance of providing, in addition to usual prenatal care, psychological support to pregnant women during stressful events that affect populations, such as the COVID-19 pandemic, has been highlighted (16).

Epidemiological studies that analyze temporal trends in diseases provide important data for evaluating strategies aimed at addressing the problem and building health actions in each region and Federative Unit. The objectives of this study were: to carry out an epidemiological analysis of orofacial cleft case distribution and temporal trend in Brazil, according the country’s regions and Federative Units, from 2010 to 2021; and to compare their proportions during the COVID-19 pandemic, in 2020 to 2021, with those of the 2010-2019 time series.

## Methods

### Design

This is an epidemiological observational time series study, using records of all babies born with orofacial clefts held on the Live Birth Information System (*Sistema de Informações sobre Nascidos Vivos* - SINASC) for the period 2010-2021.

### Setting

SINASC was implemented in 1990 to record epidemiological information about births in Brazil. This system uses the Live Birth Certificate as the base document for data collection. Abnormalities are coded in accordance with the provisions of chapter XVII of the 10th Revision of the International Statistical Classification of Diseases and Related Health Problems (ICD-10), regarding congenital malformations, deformations and chromosomal abnormalities (Q00-Q99) (2).

### Participants

The study population comprised all live births in Brazil between 2010 and 2021.

### Variables

The dependent variable was the prevalence of each type of cleft (cleft palate, cleft lip and cleft palate with cleft lip), stratified according to the Brazilian regions and Federative Units (North: Rondônia, Acre, Amazonas, Roraima, Pará, Amapá, Tocantins; Northeast: Maranhão, Piauí, Ceará, Rio Grande do Norte, Paraíba, Pernambuco, Alagoas, Sergipe, Bahia; Midwest: Mato Grosso do Sul, Mato Grosso, Goiás, Distrito Federal; South: Paraná, Santa Catarina, Rio Grande do Sul; and Southeast: São Paulo, Rio de Janeiro, Minas Gerais, Espírito Santo). 

The independent variables were the year, 2010-2021, and the period of analysis, namely the pre-COVID-19 pandemic period, 2010-2019, and the pandemic period, 2020-2021.

### Data sources and measurement

The data were collected via the Birth Monitoring Panel (*Painel de Monitoramento da Natalidade*).

We identified all children born with orofacial clefts recorded by coding on the SINASC, in accordance with ICD-10 Chapter XVII. The codes selected were: cleft palate (ICD-Q35), cleft lip (ICD-Q36) or cleft palate with cleft lip (ICD-Q37), for analysis of the longitudinal component of the time series, comprising the prevalence rates of the period 2010-2021, and comparing prevalence rates between the periods 2010-2019 and 2020-2021, for each type of cleft and total clefts.

### Bias control

Data collection was carried out in May and June 2023, with the aim of preventing the SINASC data closure deadline from interfering with the calculations of the indicators. According to Health Ministry Ordinance No. 199/2009, “data are disclosed on a preliminary basis, and later on a definitive basis, within the following deadlines: I – Between June 30 and August 30 of the year following the year of occurrence, on a preliminary basis; and II – By December 30th of the year following the year of occurrence, on a definitive basis.” (17).

### Study size

We collected data on live births in Brazil for the period 2010-2021. 

### Statistical methods

The proportions were calculated by dividing the number of births recorded with each type of orofacial clefts (cleft palate, cleft lip and cleft palate with cleft lip), in each year and region and Federative Unit, by the total number of live births for the same category and period, in the same place and period, multiplied by 10,000. We used the Prais-Winsten generalized linear regression model to assess the time series, providing the average annual change in the series values ​​(β coefficient), expressed in percentage points (p.p.) per year. 

The Durbin-Watson hypothesis test was used to check for the presence of serial autocorrelation, with values ​​close to 2 being expected. In order to check differences between categories, we used the period averages and 95% confidence intervals (95%CI), whereby these should not overlap. Average prevalence for the pandemic period, from 2020 to 2021, was compared to average prevalence for the preceding period, from 2010 to 2019, according to the Brazilian regions and 95%CI. We used a 5% significance level. 

The analyses were performed using Stata 16.0. All figures were created using the Plotly library (version 5.6.0) for Python (version 3.9.12) (18-20).

### Data access and cleaning methods

The data were available for tabulation at: https://svs.aids.gov.br/daent/centrais-de-conteudos/paineis-de-monitoramento/natalidade/anomalias-congenitas/. We used the Comma Separated Values ​​(.CSV) file extension to export them to Microsoft Excel. The data were organized and standardized on spreadsheets according to region and Federative Unit. There were no records with unknown data for the analysis we performed.

## Results

In all, 34,564,430 live births were recorded in Brazil in the period 2010-2021, with 5,407,246 (15.6%) of them occurring during the COVID-19 pandemic period from 2020 to 2021. Of the total, 23,246 presented one or more types of orofacial clefts, resulting in a prevalence rate of 6.73/10,000 live births (95%CI 6.64; 6.81). Of these, 9,633 were born with cleft palate (prevalence: 2.79/10,000; 95%CI 2.69; 2.90), 6,579 with cleft lip (1.90/10,000; 95%CI 1.83; 1.97) and 7,034 with cleft palate with cleft lip (2.04/10,000; 95%CI 1.90; 2.18).

**Figure 1:**
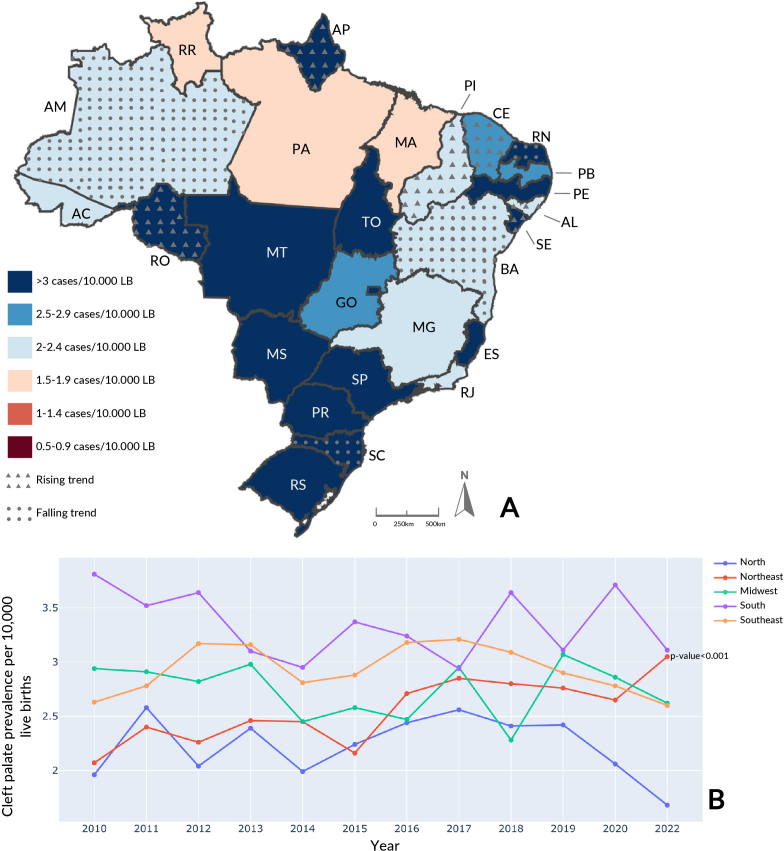
Cleft palate average prevalence and temporal trend, by Federative Unit, per 10,000 live births (LB) (A); cleft palate annual prevalence, by region, per 10,000 live births (B). Brazil, 2010-2021 (n=9,633)

The Southern region had the highest average prevalence of cleft palate: 3.35/10,000 live births (95%CI 3.15; 3.54) (Figure 1). The Federative Unit with the highest average prevalence of cleft palate was Sergipe (Northeast) with 5.06/10,000 (95%CI 2.75; 7.37). Regarding annual prevalence of cleft palate, the Federal District (Midwest) stood out in 2019 (17.44/10,000 live births) (Supplementary Table 1). Regarding the trend, cleft palate showed stability in most regions, with the exception of the Northeast, which had a rising trend (p-value<0.001), and there was also an increase in some Federative Units – Amapá and Rondônia (North) and Alagoas, Ceará, Piauí and Sergipe (Northeast) – and a reduction in others – Amazonas (North), Bahia and Rio Grande do Norte (Northeast) and Santa Catarina (South).

**Table 1:** Orofacial cleft average prevalence with 95% confidence intervals (95%CI), by region before and during the COVID-19 pandemic. Brazil, 2010-2021 (n=23,246)

Regions	2010-2019	2020-2021
	Average (95%CI)	Average (95%CI)
**Cleft palate**		
North	2.30 (2.14; 2.47)	1.87 (-0.51; 4.25)
Northeast	2.49 (2.29; 2.69)	2.85 (0.26; 5.44)
Southeast	2.98 (2.83; 3.13)	2.69 (1.55;3.83)
South	3.33 (3.11; 3.55)	3.41 (-0.36; 7.18)
Midwest	2.75 (2.55; 2.94)	2.74 (1.19; 4.29)
Brazil	2.79 (2.69; 2.90)	2.75 (2.10; 3.39)
**Cleft lip**		
North	1.59 (1.31; 1.87)	1.52 (-0.59; 3.64)
Northeast	1.66 (1.51; 1.81)	1.85 (1.37; 2.34)
Southeast	2.14 (1.99; 2.29)	1.89 (1.08; 2.69)
South	2.25 (2.11; 2.40)	1.75 (0.87; 2.63)
Midwest	1.74 (1.52; 1.96)	1.39 (0.25; 2.53)
Brazil	1.93 (1.86; 2.00)	1.78 (1.37; 2.19)
**Cleft palate with cleft lip**		
North	2.01 (1.70; 2.32)	2.70 (1.26; 4.14)
Northeast	1.44 (1.24; 1.63)	1.87 (1.36; 2.37)
Southeast	2.07 (1.93; 2.21)	2.22 (0.40; 4.04)
South	2.90 (2.71; 3.09)	3.09 (-0.65; 6.84)
Midwest	1.90 (1.76; 2.03)	2.70 (-3.49; 8.89)
Brazil	1.98 (1.84; 2.12)	2.34 (1.97; 2.70)

**Figure 2:**
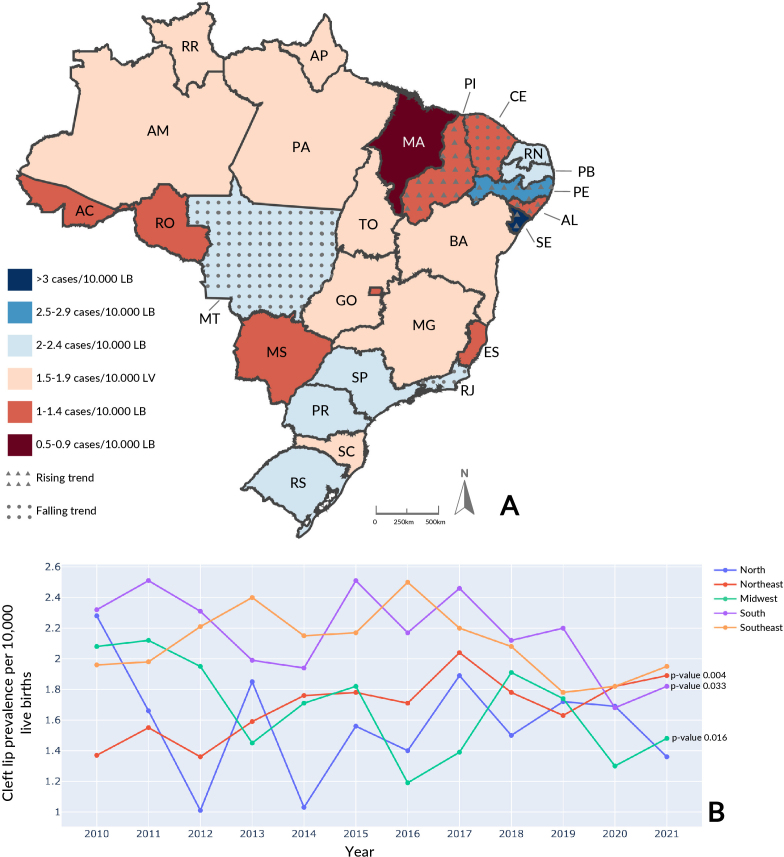
Cleft lip average prevalence and temporal trend, by Federative Unit, per 10,000 live births (LB) (A): cleft lip annual prevalence, by region, per 10,000 live births (B). Brazil, 2010-2021 (n=6,579)

The highest average cleft lip prevalence was found in the Southern region with 2.17/10,000 live births (95%CI 2.00; 2.34) (Figure 2). The Federative Unit with the highest average prevalence was Sergipe (Northeast), with 3.23/10,000 live births (95%CI 2.53; 3.94). With regard to annual prevalence, Sergipe (Northeast) stood out in 2020 (5.35/10,000 live births) (Supplementary Table 2). An increase in the temporal trend was seen in the Northeast region (p-value 0.004) and in the following Federative Units: Alagoas, Pernambuco, Piauí and Sergipe (Northeast). There was a reduction in the South (p-value 0.033) and in the Midwest (p-value 0.016) and in the following Federative Units: Ceará (Northeast), Rio de Janeiro (Southeast) and Mato Grosso (Midwest).

**Figure 3:**
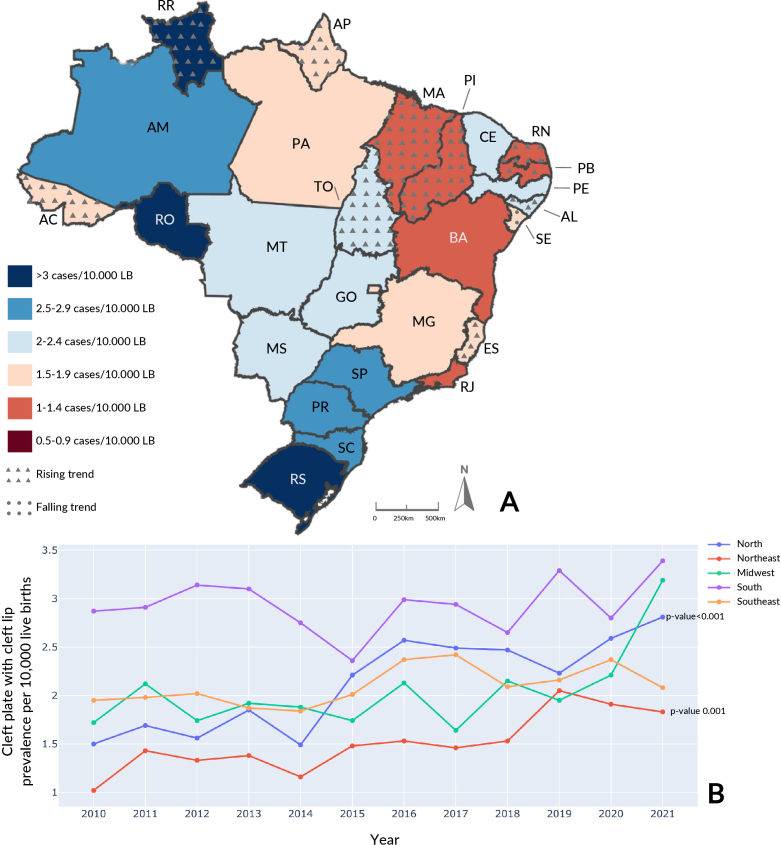
Cleft palate with cleft lip average prevalence and temporal trend, by Federative Unit, per 10,000 live births (LB) (A); annual prevalence of cleft palate with cleft lip, by region, per 10,000 live births (B). Brazil, 2010-2021 (n=7,034)

The South was the region with the highest average prevalence of cleft palate with cleft lip, with 2.93/10,000 live births (95%CI 2.75; 3.11) (Figure 3). The Federative Unit with the highest prevalence in the period was Roraima (North), with 4.31/10,000 live births (95%CI 2.69; 5.93), in particular its annual prevalence in 2016 (10.55 cases per 10,000 live births) (Supplementary Table 3). With regard to trend, there was an increase in Brazil (p-value<0.001), in the North (p-value<0.001) and Northeast (p-value 0.001) regions, in addition to the following Federative Units: Acre, Amapá, Roraima and Tocantins (North), Alagoas, Maranhão, Paraíba, Piauí and Rio Grande do Norte (Northeast) and Espírito Santo (Southeast). The only reduction was in Sergipe (Northeast).

When assessing average prevalence of orofacial clefts in the periods preceding and corresponding to the COVID-19 pandemic in the regions (Table 1), average cleft palate prevalence of 2.79/10,000 (95%CI 2.69; 2.90) from 2010 to 2019 in Brazil was similar to that for the period 2020-2021 (2.75/10,000; 95%CI 2.10; 3.39). The South recorded the highest rate for both periods with 3.33/10,000 (95%CI 3.11; 3.55) in the pre-pandemic period and 3.41/10,000 (95%CI -0.36; 7.18) in the pandemic period. The North had the lowest frequency among the country’s regions in the pre-pandemic period, 2.30/10,000 live births (95%CI 2.14; 2.47), and in the pandemic period, 1.87/10,000 (95%CI -0.51; 4.25). There were no statistically significant reductions or increases in the periods.

Average cleft lip prevalence of 1.93/10,000 was recorded in Brazil (95%CI 1.86; 2.00) from 2010 to 2019. During the COVID-19 pandemic period, it was 1.78/10,000 (95%CI 1.37; 2.19). The South had the highest proportion in the pre-pandemic period, with a prevalence rate of 2.25/10,000 (95%CI 2.11; 2.40), this being above the national average. During the pandemic period, the Southeast had the highest proportion, with a prevalence rate of 1.89/10,000 live births (95%CI 1.08; 2.69). The North had the lowest rate among the regions in the pre-pandemic period, with 1.59/10,000 live births (95%CI 1.31; 1.87). During the pandemic period, the Midwest had a prevalence rate of 1.39/10,000 live births (95%CI 0.25; 2.53). There was no significant increase or reduction in both periods.

Average prevalence of cleft palate with cleft lip from 2010 to 2019 was 1.98/10,000 live births in Brazil (95%CI 1.84; 2.12). During the COVID-19 pandemic period, there were 2.34/10,000 live births with this condition (95%CI 1.97; 2.70). The South had the highest proportion for both periods, with a prevalence rate of 2.90/10,000 live births (95%CI 2.71; 3.09) in the pre-pandemic period and 3.09/10,000 live births (95%CI -0.65; 6.84) in the COVID-19 period, this being above the national average. The Northeast had the lowest proportion among the country’s regions in the pre-pandemic and pandemic periods: 1.44/10,000 live births (95%CI 1.24; 1.63) and 1.87/10,000 live births (95%CI 1.36; 2.37). There were no significant changes between the periods.

## Discussion

This study identified that the Southern region of Brazil had the highest prevalence of the three types of orofacial clefts, and that cleft palate was the most prevalent in the period. The Northeast region showed a rising trend in the three types of orofacial clefts, as did the states of Alagoas and Piauí, located in the same region. The COVID-19 pandemic did not influence the prevalence of orofacial clefts in the Brazilian regions in the period assessed.

Among the limitations of this study, use of secondary data stood out, as it depends on the quality and completeness of the reported data. It should be emphasized that the SINASC system records abnormalities detected shortly after birth, which probably underestimates the prevalence rates recorded, especially those in the cleft palate and labiopalatal group, which depend on a more specific diagnosis by the attending physician, since they are not easily visualized in the prenatal period or during physical examination. The non-significant variations between the Brazilian regions in the number of cases of orofacial clefts during the COVID-19 pandemic period, may reflect the short post-exposure period we assessed, and the effects of the pandemic may appear in subsequent years, depending on the time of gestation and embryogenesis.

Direct comparisons of orofacial cleft prevalence between countries should be made with caution given the different methodologies used. The Brazilian prevalence rate found was lower than the rate of 14.00/10,000 in China (21) revealed by a meta-analysis using data from 1986 to 2015, and was lower than the rate of 13.80/10,000 found for low- and middle-income countries (22) by a meta-analysis covering the period from 1990 to 2014. The frequency we found was similar to a Colombian cross-sectional study (23), which revealed a rate of 6.00/10,000 live births based on data from 2009 to 2017. It was also similar to the rate of 5.00 cases/10,000 live births found by a Nigerian study (24) based on data from September 2006 to June 2011. This discrepancy may be due to the different periods analyzed, racial, cultural and economic diversity, environmental exposures and differences in pregnancy care programs, in addition to differences in the operational characteristics of congenital abnormality surveillance programs in each of these countries (1,8,21). 

When comparing the result of this study with the national prevalence in different periods, such as 2005-2016 (6) when it was 5.10/10,000 live births; 2010-2019 (2) when it was 6.20/10,000; 2008-2017 (24) when it was 5.20/10,000; and 1999-2020 (7) when it was 5.16/10,000, the results suggest a slight increase, if only the most recent data are analyzed, which may be related to the improvement in case recording by health professionals on SINASC in recent years. This is because, from 2011 onwards, there was an increase in the number of characters for recording congenital abnormalities in field 34 of the Live Birth Certificate. As of 2018, Law No. 13,685 made it compulsory to report congenital abnormalities, resulting the reporting and detailing of all congenital abnormalities identified in newborns (2).

Cleft palate with cleft lip is the most prevalent type of orofacial cleft in international studies (25,26) and in previous national studies (7,27,28), unlike what we found in this study, in which cleft palate was the most frequent in the period. This discrepancy between the findings has already been observed previously by the Brazilian Ministry of Health (2) and in analyses of the Latin American Collaborative Study of Congenital Malformations (8,29), which suggested that this occurs due to SINASC using ICD-10. The shortcomings of ICD-10 for coding the orofacial cleft types is explained in the literature, and such errors are expected in any records that use this coding system. When compared to the previous system (ICD-9), ICD-10, as used on SINASC does not have modifiers for severity or clinical subtypes, which means that it lacks accuracy and precision for adequately describing orofacial clefts (29).

High underreporting of cleft palate with cleft lip (Q37) could be explained by the use of the cleft lip code (Q36) and the cleft palate code (Q35) to specify a case of cleft palate with cleft lip, as this is a common error found on SINASC (29). Misuse of codes Q38.0, Q38.5 and Q38.6 to code orofacial clefts has been noted. This could contribute to this discrepancy in prevalence (29), converging with the difficulty of many Brazilian maternity hospitals in establishing an accurate diagnosis before completing the Live Birth Certificate, which is another hypothesis that has been highlighted (8).

Among the Brazilian regions, the South had the highest prevalence for all types of orofacial clefts, whereby occurrence was higher than the Brazilian national average in the period, converging with previous national data (2,6,7). This result may be related to better case identification and recording in this region (2,8,29). An example of this better reporting is the pilot project focused on surveillance of congenital abnormalities, carried out since 2020, in the state of Rio Grande do Sul (South) (2), which includes training mother and child health care teams at sentinel hospitals to identify congenital abnormalities.

There is variation in the lowest prevalence rate regarding type of cleft: sometimes it is found in the Northeast, sometimes it is found in the North (6,24). This may reflect the presence of inequalities between Brazilian regions in relation to technological resources, training of health professionals and training of health service managers to analyze health information (7,8). The Federative Units and regions with the greatest technological and health challenges may be those with the lowest prevalence over the period.

The temporal trend, in the period studied, in the Federative Units of the South and in almost all those of the Southeast, presented a certain stability, not showing a trend of significant increase or reduction, converging with the stationary trend in the state of São Paulo (Southeast) between 2008 and 2019 (27). This can be explained by lower variability in the recording of congenital abnormalities on SINASC in these regions and the better quality of case reporting (2,8).

As in 2019 (6), the Northeast region showed a rising trend in the period, which was followed by the states of Alagoas and Piauí in that region, for of all types of orofacial clefts. This may reflect the recovery of epidemiological surveillance in these states and regions, correcting previous underestimation there (8), which converges with the improvements made in the Brazilian National Health System and in epidemiological surveillance in recent years, especially in the face of health emergencies (2,8).

An example of this is the state of Pernambuco (Northeast). Through its Center for Strategic Information on Health Surveillance, in view of the increase in the occurrence of microcephaly as a result of Zika virus, the state carried out an assessment of the event and put in place its response process to that Public Health Emergency, which was triggered internationally (2). It is suggested that these investments in improvements in health surveillance and congenital abnormalities in general, as is already occurring in Pernambuco (Northeast) and Rio Grande do Sul (South), should be continued and expanded to other states and regions, aiming at better health planning and care for congenital abnormalities throughout the national territory.

Few differences were found regarding the COVID-19 pandemic. Perhaps due to the short period of time post-exposure we assessed, from 2020 to 2021, or due to the ecological approach of our research, which did not assess specific cases of maternal infection during pregnancy, no Brazilian region showed a significant increase or reduction in the prevalence of any type of orofacial clefts when compared to the period prior to the pandemic (2010-2019).

There is disagreement in the literature as to whether or not COVID-19 may be a factor associated with the development of orofacial clefts. Even with the high accuracy of prenatal ultrasound in assessing the type of orofacial cleft (detection of the correct type of cleft in 88.90% of cases [73.70%-93.70%]), there are regional discrepancies in prenatal care and, consequently, in the diagnosis of congenital abnormalities during pregnancy (2,30). These differences may have worsened in the pandemic period, in which attention and investments were focused on tackling the health crisis, basic services were interrupted and people stopped routinely seeking health services.

Among the Brazilian regions, the South had the highest prevalence of the three types of orofacial clefts. The Northeast and its states of Alagoas and Piauí showed a rising trend for the three types of cleft, unlike other regions and Federative Units, where the trend was stationary or with increases/decreases in just one type of orofacial cleft.

Isolated cleft palate was the most common type found in this study. The COVID-19 pandemic did not influence prevalence of orofacial clefts in the short post-exposure period assessed. These findings contribute to the creation of public health policies and encourage discussion about the importance of improvements in data capture on secondary databases, such as SINASC. Clarifying the epidemiology of diseases at the national level is essential for ensuring full inclusion of children with these conditions in society and their quality of life, enabling better guidance for prevention policies, early diagnosis, care and multidisciplinary treatment of cases, in addition to guiding resource distribution. 

## Data Availability

The database and the analysis codes used in this research are available at: https://docs.google.com/spreadsheets/d/1H7jQxqmVio-9LW2JsvSLSXmR0f8shx3S3VKtwhK0M6U/edit?usp=sharing. The repository is available at: https://repositorio.animaeducacao.com.br/items/5a63e8f7-bbe6-46f3-a418-2d48a6961e7d/.
