# Supplementary material for: Orofacial clefts in newborns in Brazil: a time series study, 2010-2021
Source: Epidemiol Serv Saude. 2025 May 12;34:e20240027. doi: 10.1590/S2237-96222025v34e20240027.en (PMC12077537; doi:10.1590/S2237-96222025v34e20240027.en)
Supplement: Translation

# Translation: Fendas orofaciais em recém-nascidos no Brasil: estudo de série temporal, 2010-2021

**DOI:** 10.1590/S2237-96222025v34e20240027.pt

**Authors:** Karoline Machado Vieira, Verônica Canarim Menezes, Augusto César Cardoso-dos-Santos, Ana Luiza Meneguci Moreira Franco, Flavia Martinez de Carvalho, Lavinia Schuler Faccini, Betine Pinto Moehlecke Iser

**Affiliations:** 1 Universidade do Sul de Santa Catarina, Curso de Medicina, Tubarão, SC, Brasil; 2 Ministério da Saúde, Departamento de Ciência e Tecnologia, DF, Brasil; 3 Universidade Federal do Rio de Janeiro, Programa de Pós-Graduação em Genética, Rio de Janeiro, RJ, Brasil; 4 Instituto Oswaldo Cruz, Laboratório de Epidemiologia de Malformações Congênitas, Rio de Janeiro, RJ, Brasil; 5 Universidade Federal do Rio Grande do Sul, Programa de Pós-Graduação em Genética e Biologia Molecular, Porto Alegre, RS, Brasil; 6 Universidade do Sul de Santa Catarina, Programa de Pós-Graduação em Ciências da Saúde, Tubarão, SC, Brasil

**Keywords:** Covid-19, Fenda Labial, Fissura Palatina, Estudos de Séries Temporais, Brasil

## Abstract

**Objetivo:**

Analisar a distribuição dos casos e a tendência temporal de fendas orofaciais (fenda palatina, fenda labial e fenda labial com fenda palatina) no Brasil, segundo regiões e unidades da Federação, nos últimos 12 anos, além de comparar proporções durante a pandemia de covid-19, de 2020 a 2021, com a série histórica anterior, de 2010 a 2019.

**Métodos:**

Trata-se de estudo epidemiológico de série temporal, utilizando registros de todos os nascidos com fendas orofaciais, do Sistema de Informações sobre Nascidos Vivos, no período 2010-2021. Prevalências foram calculadas segundo ano, regiões e unidades da Federação. A análise da série temporal foi realizada pelo modelo linear generalizado de Prais-Winsten.

**Resultados:**

Foram registrados 34.564.430 nascidos vivos no período. A prevalência nacional de fendas orofaciais foi 6,73/10 mil nascidos vivos (intervalo de confiança de 95% [IC95% 6,64; 6,81]). O Sul apresentou a maior taxa no período para todos os tipos de fendas orofaciais. A região Nordeste e as unidades da Federação Alagoas e Piauí apresentaram tendência crescente no período nos três tipos de fendas orofaciais. Outras regiões apresentaram tendência estacionária ou aumentos/diminuições em apenas um tipo de fenda. Comparando o período pré-pandemia com o período da pandemia, não houve mudanças significativas na prevalência das regiões brasileiras.

**Conclusão:**

Entre as regiões do Brasil, nos três tipos de fenda, o Sul apresentou a maior prevalência, e o Nordeste, tendência ascendente. Entre as unidades da Federação, Alagoas e Piauí apresentaram aumento nos três tipos de fenda. A pandemia de covid-19 não influenciou nas prevalências no período analisado.

## Introdução

As fendas orofaciais são anomalias congênitas craniofaciais que acontecem por defeitos de formação dos palatos primário e secundário no início da formação do embrião, acometendo, com variável extensão, lábios, cavidade oral e nasal (1). Considerando os processos embrionário e patogênico envolvidos, são divididas em fenda palatina isolada e fenda labial com ou sem fenda palatina e podem ocorrer de forma isolada (70,0% dos casos) ou associada a uma ampla gama de síndromes (30,0% dos casos) (1-3). 

As fendas orofaciais podem gerar prejuízos dentários, na aparência, na fala, na audição, na cognição e nos aspectos psicossociais (1,2,9). Tais fendas podem atrapalhar no estado nutricional do recém-nascido, uma vez que influenciam na habilidade natural de se alimentar, nos mecanismos de sucção e deglutição (2,10,11). Observou-se que, em lactentes com a presença de fendas orofaciais, a chance de mortalidade era 2,07 vezes maior do que naqueles sem essa anomalia congênita (12). Os impactos da fenda orofacial requerem cuidados multidisciplinares, incluindo assistência clínica e cirúrgica, desde o nascimento até a idade adulta, já que podem levar a resultados adversos duradouros para a saúde e a integração social (2). As fendas orofaciais podem gerar prejuízos dentários, na aparência, na fala, na audição, na cognição e nos aspectos psicossociais (1,2,9). Tais fendas podem atrapalhar no estado nutricional do recém-nascido, uma vez que influenciam na habilidade natural de se alimentar, nos mecanismos de sucção e deglutição (2,10,11). Observou-se que, em lactentes com a presença de fendas orofaciais, a chance de mortalidade era 2,07 vezes maior do que naqueles sem essa anomalia congênita (12). Os impactos da fenda orofacial requerem cuidados multidisciplinares, incluindo assistência clínica e cirúrgica, desde o nascimento até a idade adulta, já que podem levar a resultados adversos duradouros para a saúde e a integração social (2).

A etiologia das fendas orofaciais é complexa e multifatorial com aspectos genéticos e ambientais envolvidos na sua formação (1,3). Consumo de bebidas alcoólicas, exposição ao fumo (ativa ou passivamente) e exposição a fenitoína, ácido valproico e talidomida, no primeiro trimestre da gestação, são os principais fatores ambientais associados à anomalia congênita (1,2,12). A ocorrência de febre materna e infecções já foi associada ao desenvolvimento das fendas orofaciais, assim como eventos estressantes durante a vida e o período periconcepcional e o risco de fendas orofaciais na prole (13,14). 

Questiona-se se eventos estressores e outras infecções, como é o caso da covid-19, doença causada pelo coronavírus SARS-CoV-2, poderiam influenciar na ocorrência das fendas orofaciais, visto que geram também grande efeito psicológico e estressor, além de sintomas que podem se assemelhar a um resfriado comum, com a presença da febre (15). A ocorrência de fendas orofaciais pode estar associada à exposição materna ao estresse ao longo da vida e ao medo da covid-19 em particular, mas, sem efeito direto da própria infecção (16). Destaca-se a importância de fornecer, além dos cuidados pré-natais habituais, apoio psicológico às gestantes durante eventos estressantes que afetam populações, como a pandemia de covid-19 (16).

Estudos epidemiológicos que analisam tendências temporais dos agravos fornecem dados importantes para a avaliação das estratégias visando ao enfrentamento do problema e à construção de ações em saúde em cada região e unidades da Federação. Os objetivos deste estudo foram: realizar a análise epidemiológica da distribuição dos casos e a tendência temporal das fendas orofaciais no Brasil, segundo regiões e unidades da Federação, nos últimos 12 anos; e comparar as proporções durante a pandemia de covid-19, em 2020 a 2021, com as da série histórica de 2010 a 2019.

## Métodos

### Delineamento

Trata-se de estudo epidemiológico, observacional, de série temporal, utilizando registros de todos os nascidos com fendas orofaciais, do Sistema de Informações sobre Nascidos Vivos (Sinasc), no período 2010-2021.

### Contexto

O Sinasc foi implantado em 1990 para registro de informações epidemiológicas sobre os nascimentos no país. Esse sistema tem a Declaração de Nascido Vivo como documento-base para coleta de dados. As anomalias são codificadas atendendo ao disposto no capítulo XVII da Classificação Estatística Internacional de Doenças e Problemas Relacionados à Saúde, na sua décima revisão (CID-10), que discorre sobre as malformações congênitas, deformidades e anomalias cromossômicas (Q00-Q99) (2).

### Participantes

A população do estudo compreendeu todos os nascidos vivos no país entre 2010 e 2021.

### Variáveis

A variável dependente foi a prevalência de cada tipo de fenda (fenda palatina, fenda labial e fenda labial com fenda palatina), estratificada segundo região e unidades da Federação do Brasil (Norte: Rondônia, Acre, Amazonas, Roraima, Pará, Amapá, Tocantins; Nordeste: Maranhão, Piauí, Ceará, Rio Grande do Norte, Paraíba, Pernambuco, Alagoas, Sergipe, Bahia; Centro-Oeste: Mato Grosso do Sul, Mato Grosso, Goiás, Distrito Federal; Sul: Paraná, Santa Catarina, Rio Grande do Sul; e Sudeste: São Paulo, Rio de Janeiro, Minas Gerais, Espírito Santo). 

As variáveis independentes foram o ano, 2010-2021, e o período de análise, sendo o período pré-pandemia de covid-19, 2010-2019, e o período concorrente a pandemia, 2020-2021.

### Fontes de dados e mensuração

Os dados foram coletados através do Painel de Monitoramento da Natalidade.

Foram identificados todos os nascidos com fendas orofaciais registrados, codificadas no Sinasc, atendendo ao capítulo XVII da CID-10. Os códigos selecionados foram: fenda palatina (CID-Q35), fenda labial (CID-Q36) ou fenda labial com fenda palatina (CID-Q37), para análise do componente longitudinal de série temporal, compreendendo as prevalências do período 2010-2021, e comparadas as prevalências entre os períodos 2010-2019 e 2020-2021, de cada tipo de fenda e do total.

### Controle de viés

A coleta de dados foi realizada em maio e junho de 2023, com o intuito de evitar que o tempo de encerramento dos dados no Sinasc interferisse nos cálculos dos indicadores. Conforme a Portaria nº 199/2009, “os dados são divulgados em caráter preliminar, e posteriormente em caráter definitivo, nos seguintes prazos: I – Entre 30 de junho e 30 de agosto do ano subsequente ao ano de ocorrência, em caráter preliminar; e II – Até 30 de dezembro do ano subsequente ao ano de ocorrência, em caráter oficial.” (17).

### Tamanho do estudo

Foram captados dados dos nascidos vivos do Brasil de 2010 a 2021. 

### Métodos estatísticos

As proporções foram calculadas dividindo-se o número de nascimentos registrados com cada tipo de fendas orofaciais (fenda palatina, fenda labial e fenda labial com fenda palatina), em cada ano e região e unidades da Federação, pelo total de nascidos vivos para a mesma categoria e período, no mesmo local e período, multiplicado por 10 mil. Para avaliação da série temporal, utilizou-se o modelo de regressão linear generalizada de Prais-Winsten, fornecendo a variação média anual dos valores das séries (coeficiente β), expresso em pontos percentuais (p.p.) ao ano. 

O teste de hipóteses de Durbin-Watson foi útil para checar a presença de autocorrelação serial, sendo esperados valores próximos de 2. Para verificar diferenças entre as categorias, consideraram-se a média do período e os intervalos de confiança de 95% (IC95%), não devendo estes sobrepor-se. A prevalência média do período da pandemia, de 2020 a 2021, foi comparada à prevalência média do período anterior, de 2010 a 2019, segundo regiões do Brasil e IC95%. O nível de significância foi 5,0%. 

As análises foram realizadas utilizando-se o programa Stata 16.0. Todas as figuras foram elaboradas utilizando a biblioteca Plotly (versão 5.6.0) para Python (versão 3.9.12) (18-20).

### Acesso aos dados e métodos de limpeza

Dados disponíveis para tabulação no endereço eletrônico: https://svs.aids.gov.br/daent/centrais-de-conteudos/paineis-de-monitoramento/natalidade/anomalias-congenitas/. Foi utilizada a extensão Comma Separated Values (.CSV) para a plataforma Microsoft Excel. Os dados foram organizados e padronizados em planilhas segundo a região e as unidades da Federação. Não houve registros ignorados para a análise realizada.

## Resultados

Foram registrados 34.564.430 nascidos vivos no Brasil no período 2010-2021, sendo 5.407.246 (15,6%) deles no período da pandemia de covid-19 de 2020 a 2021. Do total, 23.246 apresentaram um ou mais tipos de fendas orofaciais, resultando na prevalência de 6,73/10 mil nascidos vivos (IC95% 6,64; 6,81). Desses, 9.633 nasceram com fenda palatina (prevalência: 2,79/10 mil; IC95% 2,69; 2,90), 6.579 com fenda labial (1,90/10 mil; IC95% 1,83; 1,97) e 7.034 com fenda labial com fenda palatina (2,04/10 mil; IC95% 1,90; 2,18).

A região Sul apresentou a maior prevalência média de fenda palatina: 3,35/10 mil nascidos vivos (IC95% 3,15; 3,54) (Figura 1). A unidade da Federação com a maior prevalência média de fenda palatina foi Sergipe (Nordeste) com 5,06/10 mil (IC95% 2,75; 7,37). Na prevalência anual das fendas palatinas, destacou-se o Distrito Federal (Centro-Oeste) em 2019 (17,44/10 mil nascidos vivos) (Tabela suplementar 1). Na tendência, as fendas palatinas apresentaram estabilidade na maioria das regiões, com exceção do Nordeste que teve tendência crescente (p-valor<0,001), além de aumento em algumas unidades da Federação – Amapá e Rondônia (Norte) e Alagoas, Ceará, Piauí e Sergipe (Nordeste) – e redução em outras – Amazonas (Norte), Bahia e Rio Grande do Norte (Nordeste) e Santa Catarina (Sul).

**Figura 1:** Prevalência média de fenda palatina e tendência temporal, por unidade da federação, por 10 mil nascidos vivos (NV) (A); frevalência anual de fenda palatina, por região, por 10 mil nascidos vivos (B). Brasil, 2010-2021 (n=9.633)

Observou-se a maior prevalência média de fendas labiais na região Sul com 2,17/10 mil nascidos vivos (IC95% 2,00; 2,34) (Figura 2). A unidade da Federação com maior prevalência média foi Sergipe (Nordeste), com 3,23/10 mil nascidos vivos (IC95% 2,53; 3,94). Na prevalência anual, evidenciou-se Sergipe (Nordeste) em 2020 (5,35/10 mil nascidos vivos) (Tabela suplementar 2). Observou-se aumento na tendência temporal na região Nordeste (p-valor 0,004) e nas unidades da Federação Alagoas, Pernambuco, Piauí e Sergipe (Nordeste). Houve redução no Sul (p-valor 0,033) e no Centro-Oeste (p-valor 0,016) e nas unidades da Federação Ceará (Nordeste), Rio de Janeiro (Sudeste) e Mato Grosso (Centro-Oeste).

**Figura 2:** Prevalência média de fenda labial e tendência temporal, por unidade da federação, por 10 mil nascidos vivos (NV) (A); prevalência anual de fenda labial, por região, por 10 mil nascidos vivos (B). Brasil, 2010-2021 (n=6.579)

O Sul foi a região que apresentou maior prevalência média de fenda labial com fenda palatina, com 2,93/10 mil nascidos vivos (IC95% 2,75; 3,11) (Figura 3). A unidade da Federação com a maior prevalência no período foi Roraima (Norte), com 4,31/10 mil nascidos vivos (IC95% 2,69; 5,93), destaque para a prevalência anual de 2016 (10,55 casos por 10 mil nascidos vivos) (Tabela suplementar 3). Na tendência, verificou-se aumento no Brasil (p-valor<0,001), nas regiões Norte (p-valor<0,001) e Nordeste (p-valor 0,001), além das unidades da Federação Acre, Amapá, Roraima e Tocantins (Norte), Alagoas, Maranhão, Paraíba, Piauí e Rio Grande do Norte (Nordeste) e Espírito Santo (Sudeste). Houve redução apenas em Sergipe (Nordeste).

**Figura 3:** Prevalência média de fenda labial com fenda palatina e tendência temporal, por unidade da federação, por 10 mil nascidos vivos (NV) (A); prevalência anual de fenda labial com fenda palatina, por região, por 10 mil nascidos vivos (B). Brasil, 2010-2021 (n=7.034)

Ao avaliar a prevalência média das fendas orofaciais no período anterior e concorrente à pandemia de covid-19 nas regiões (Tabela 1), a média de fendas palatinas de 2010 a 2019, de 2,79/10 mil (IC95% 2,69; 2,90), no Brasil foi similar àquela do período 2020-2021 (2,75/10 mil; IC95% 2,10; 3,39). O Sul registrou a maior taxa para ambos os períodos com 3,33/10 mil (IC95% 3,11; 3,55) no pré-pandemia e 3,41/10 mil (IC95% -0,36; 7,18) na pandemia. O Norte apresentou a menor frequência entre as regiões no período pré-pandemia, 2,30/10 mil nascidos vivos (IC95% 2,14; 2,47), e na pandemia, 1,87/10mil (IC95% -0,51; 4,25). Não se verificaram reduções ou aumentos estatisticamente significantes nos períodos.

**Tabela 1:** Prevalência média com intervalo de confiança de 95% (IC95%) de fendas orofaciais por região antes e durante a pandemia de covid-19. Brasil, 2010-2021 (n=23.246)

Regiões	2010-2019	2020-2021
	Média (IC95%)	Média (IC95%)
**Fendas palatinas**		
Norte	2,30 (2,14; 2,47)	1,87 (-0,51; 4,25)
Nordeste	2,49 (2,29; 2,69)	2,85 (0,26; 5,44)
Sudeste	2,98 (2,83; 3,13)	2,69 (1,55;3,83)
Sul	3,33 (3,11; 3,55)	3,41 (-0,36; 7,18)
Centro-Oeste	2,75 (2,55; 2,94)	2,74 (1,19; 4,29)
Brasil	2,79 (2,69; 2,90)	2,75 (2,10; 3,39)
**Fendas labiais**		
Norte	1,59 (1,31; 1,87)	1,52 (-0,59; 3,64)
Nordeste	1,66 (1,51; 1,81)	1,85 (1,37; 2,34)
Sudeste	2,14 (1,99; 2,29)	1,89 (1,08; 2,69)
Sul	2,25 (2,11; 2,40)	1,75 (0,87; 2,63)
Centro-Oeste	1,74 (1,52; 1,96)	1,39 (0,25; 2,53)
Brasil	1,93 (1,86; 2,00)	1,78 (1,37; 2,19)
**Fendas labiais com fendas palatinas**		
Norte	2,01 (1,70; 2,32)	2,70 (1,26; 4,14)
Nordeste	1,44 (1,24; 1,63)	1,87 (1,36; 2,37)
Sudeste	2,07 (1,93; 2,21)	2,22 (0,40; 4,04)
Sul	2,90 (2,71; 3,09)	3,09 (-0,65; 6,84)
Centro-Oeste	1,90 (1,76; 2,03)	2,70 (-3,49; 8,89)
Brasil	1,98 (1,84; 2,12)	2,34 (1,97; 2,70)

Foi registrada a prevalência média de 1,93/10 mil no Brasil (IC95% 1,86; 2,00) de 2010 a 2019 quanto à fenda labial. No período de pandemia de covid-19, foi 1,78/10 mil (IC95% 1,37; 2,19). O Sul obteve a maior proporção no período pré-pandemia, com prevalência de 2,25/10 mil (IC95% 2,11; 2,40), acima da média nacional. No período da pandemia, foi o Sudeste com prevalência de 1,89/10 mil nascidos vivos (IC95% 1,08; 2,69). O Norte apresentou a menor taxa entre as regiões no período pré-pandemia, com 1,59/10 mil nascidos vivos (IC95% 1,31; 1,87). No período da pandemia, o Centro-Oeste apresentou a prevalência de 1,39/10 mil nascidos vivos (IC95% 0,25; 2,53). Não houve aumento ou redução significativa nos dois períodos.

A prevalência média de fenda labial com fenda palatina de 2010 a 2019 foi 1,98/10 mil nascidos vivos no Brasil (IC95% 1,84; 2,12). No período da pandemia de covid-19, foi 2,34/10 mil nascidos vivos (IC95% 1,97; 2,70). O Sul obteve a maior proporção para ambos os períodos, com prevalência de 2,90/10 mil nascidos vivos (IC95% 2,71; 3,09) no período pré-pandemia e 3,09/10 mil nascidos vivos (IC95% -0,65; 6,84) na pandemia de covid-19, acima da média nacional. O Nordeste apresentou a menor proporção entre as regiões nos períodos pré-pandemia e pandêmico: 1,44/10 mil nascidos vivos (IC95% 1,24; 1,63) e 1,87/10 mil nascidos vivos (IC95% 1,36; 2,37). Não foram verificadas alterações significantes entre os períodos.

## Discussão

Este estudo identificou que a região Sul apresentou a maior prevalência nos três tipos de fendas orofaciais, e a fenda palatina foi a mais prevalente no período. A região Nordeste apresentou tendência ascendente nos três tipos de fendas orofaciais, assim como as unidades da Federação Alagoas e Piauí, da mesma região. A pandemia de covid-19 não influenciou nas prevalências das fendas orofaciais entre as regiões brasileiras no período avaliado.

Entre as limitações deste estudo, destacou-se o uso de dados secundários, dependente da qualidade e completitude dos dados notificados. Ressalta-se que o Sinasc registra as anomalias detectadas logo após o nascimento, o que provavelmente subestime as prevalências registradas, sobretudo no grupo da fenda palatina e labiopalatal, que dependem de diagnóstico mais específico pelo profissional assistente, uma vez que não são facilmente visualizadas no período pré-natal e no exame físico. As variações não significantes do número de casos de fendas orofaciais durante o período da pandemia de covid-19, entre as regiões, podem refletir o curto período pós-exposição avaliado, podendo os efeitos da pandemia aparecerem nos anos subsequentes, a depender do momento da gestação e embriogênese.

Comparações diretas entre as prevalências das fendas orofaciais entre os países devem ser cautelosas tendo em vista as diferentes metodologias utilizadas. A prevalência brasileira encontrada foi menor que a da China (21), vista em uma metanálise com dados de 1986 a 2015, de 14,00/10 mil, e foi menor que a de países de baixa e média renda (22), vista em metanálise de 1990 a 2014, de 13,80/10 mil. A frequência vista foi semelhante a um estudo transversal colombiano (23), com dados de 2009 a 2017, de 6,00/10 mil nascidos vivos, e a um nigeriano (24), com dados de setembro de 2006 a junho de 2011, de 5,00 casos/10 mil nascidos vivos. Essa discrepância pode ser devida aos diferentes períodos analisados, à diversidade racial, cultural e econômica, às exposições ambientais e às diferenças nos programas de atendimento às gestantes, além das diferenças nas características operacionais dos programas de vigilância de anomalias congênitas em cada país (1,8,21). 

Quando comparado o resultado deste estudo com a prevalência nacional em diferentes períodos, a exemplo de 2005-2016 (6) de 5,10/10 mil nascidos vivos; 2010-2019 (2) de 6,20/10 mil; 2008-2017 (24) de 5,20/10 mil; e 1999-2020 (7) de 5,16/10 mil, os resultados sugerem leve aumento, se analisados apenas os dados mais recentes, podendo estar relacionado à melhoria na captação dos casos pelos profissionais de saúde por meio do Sinasc nos últimos anos. Isso porque, a partir de 2011, houve aumento no número de caracteres para o registro das anomalias congênitas no campo 34 da Declaração de Nascidos Vivos. A partir de 2018, por meio da Lei nº 13.685, a notificação das anomalias congênitas passou a ter caráter compulsório, permitindo a notificação e o detalhamento de todas as anomalias congênitas identificadas no recém-nascido (2).

A fenda labial com fenda palatina é o tipo mais prevalente de fendas orofaciais em estudos internacionais (25,26) e em estudos nacionais anteriores (7,27,28), diferentemente do encontrado neste estudo, no qual a fenda palatina foi a mais frequente no período. Essa discrepância entre os achados já foi percebida anteriormente pelo Ministério da Saúde do Brasil (2) e em análises do Estudo Colaborativo Latino-Americano de Malformações Congênitas (8,29), que sugeriram que isso ocorre devido à utilização da CID-10 pelo Sinasc. A deficiência desse sistema para codificar os tipos das fendas orofaciais é explicada na literatura, sendo tais erros esperados em qualquer registro que utilize esse sistema de codificação. O Sinasc, quando comparado com o sistema anterior, CID-9, não possui modificadores para gravidade ou subtipos clínicos, o que significa que falta exatidão e precisão para descrever adequadamente as fendas orofaciais (29). 

A alta subnotificação de fenda labial com fenda palatina (Q37) poderia ser explicada pela utilização do código fenda labial (Q36) e com o código da fenda palatina (Q35) para especificar um caso de fenda labial com fenda palatina, pois esse é um erro comum encontrado no Sinasc (29). Foi percebida a utilização indevida dos códigos Q38.0, Q38.5 e Q38.6 para codificar fendas orofaciais. Isso poderia contribuir para essa discrepância de prevalências (29) convergindo com a dificuldade de muitas maternidades brasileiras estabelecerem um diagnóstico preciso antes do registro na Declaração de Nascido Vivo, outra hipótese destacada (8).

Entre as regiões brasileiras, o Sul apresentou a maior prevalência para todos os tipos, com ocorrência maior que a média nacional brasileira no período, convergindo com dados nacionais anteriores (2,6,7). Tal resultado pode ter relação com o melhor registro e captação dos casos nessa região (2,8,29). Um exemplo dessa melhor notificação é o projeto-piloto focado na vigilância de anomalias congênitas, realizado desde 2020, no Rio Grande do Sul (Sul) (2), que inclui capacitação das equipes materno-infantis de hospitais sentinelas para a identificação de anomalias congênitas.

Há variação na menor prevalência quanto ao tipo de fenda: ora é o Nordeste, ora é o Norte (6,24). Isso pode refletir a presença de desigualdades entre as regiões brasileiras em relação aos recursos tecnológicos, à formação dos profissionais de saúde e à capacitação dos gestores para analisar informações de saúde (7,8). As unidades da Federação e as regiões com maiores desafios tecnológicos e em saúde podem ser as que apresentam as menores prevalências ao longo do período.

A tendência temporal, no período estudado, nas unidades da Federação do Sul e em quase todas do Sudeste apresentou certa estabilidade, não mostrando tendência de aumento ou redução significativas, convergindo com a tendência estacionária no estado de São Paulo (Sudeste) entre 2008 e 2019 (27). Isso pode ser explicado pela menor variabilidade no registro de anomalias congênitas no Sinasc nessas regiões e pela melhor qualidade da notificação (2,8). 

Assim como em 2019 (6), o Nordeste apresentou tendência ascendente no período, acompanhada pelas suas unidades da Federação Alagoas e Piauí, para o desenvolvimento de todos os tipos de fendas orofaciais. Isso pode refletir a recuperação da vigilância epidemiológica nesses estados e regiões, corrigindo a anterior subestimação nesses locais (8), o que converge com as melhorias feitas no Sistema Único de Saúde e na vigilância epidemiológica nos últimos anos, principalmente diante das emergências em saúde (2,8).

Um exemplo disso é Pernambuco (Nordeste). Através do Centro de Informações Estratégicas em Vigilância em Saúde, com o aumento da ocorrência da microcefalia em decorrência do Zika vírus, o estado realizou a avaliação do evento e desencadeou o processo de resposta à Emergência em Saúde Pública, deflagrada internacionalmente (2). Sugere-se que esses investimentos em melhorias na vigilância em saúde e das anomalias congênitas em geral, como já ocorre em Pernambuco (Nordeste) e no Rio Grande do Sul (Sul), devem ser continuados e ampliados para outros estados e regiões, visando ao melhor planejamento em saúde e à assistência das anomalias congênitas em todo território nacional.

Poucas diferenças foram observadas referentes à pandemia de covid-19. Talvez pelo curto espaço de tempo pós-exposição avaliado, de 2020 a 2021, ou pela abordagem ecológica da pesquisa, que não avaliou casos específicos de infecção materna durante a gravidez, nenhuma região brasileira apresentou aumento ou redução significativa na prevalência em nenhum tipo de fendas orofaciais quando comparado com o período anterior, de 2010 a 2019, à pandemia. 

Há divergências na literatura se a covid-19 pode ou não ser um fator associado ao desenvolvimento das fendas orofaciais. Mesmo com a alta precisão da ultrassonografia pré-natal na avaliação do tipo de fenda orofacial (detecção do tipo correto de fenda de 88,90% [73,70%-93,70%]), há discrepâncias regionais na assistência pré-natal e, consequentemente, no diagnóstico de anomalias congênitas no período gestacional (2,30). Tais diferenças podem ter se agravado no período concorrente à pandemia, no qual as atenções e os investimentos estavam voltados ao enfrentamento da crise sanitária, os serviços básicos foram interrompidos e as pessoas deixaram de procurar serviços de saúde de forma rotineira.

O Sul apresentou a maior prevalência nos três tipos de fendas orofaciais entre as regiões brasileiras. O Nordeste e as unidades da Federação Alagoas e Piauí apresentaram tendência crescente para os três tipos de fenda, diferentemente das outras regiões e unidades da Federação, onde a tendência foi estacionária ou com aumentos/diminuições em apenas um tipo de fenda orofacial. 

A fenda palatina isolada foi a mais frequente neste estudo. A pandemia de covid-19 não influenciou na prevalência de fendas orofaciais, no curto período pós-exposição avaliado. Estes achados contribuem para a criação de políticas públicas em saúde e fomentam a discussão sobre a importância das melhorias na captação dos dados em bases secundárias, como o Sinasc. O esclarecimento da epidemiologia dos agravos no âmbito nacional é essencial para assegurar a total inclusão dessas crianças na sociedade e sua qualidade de vida, podendo nortear melhor as políticas de prevenção, o diagnóstico precoce, a assistência e o tratamento multidisciplinar dos casos, além de orientar a distribuição de recursos. 

## Data Availability

O banco de dados e os códigos de análise utilizados na pesquisa estão disponíveis em: https://docs.google.com/spreadsheets/d/1H7jQxqmVio-9LW2JsvSLSXmR0f8shx3S3VKtwhK0M6U/edit?usp=sharing. O repositório está disponível em: https://repositorio.animaeducacao.com.br/items/5a63e8f7-bbe6-46f3-a418-2d48a6961e7d/.
